# Bromido(2-{1-[2-(piperidin-1-yl)ethyl­imino]­eth­yl}phenolato)copper(II)

**DOI:** 10.1107/S1600536810026711

**Published:** 2010-07-10

**Authors:** Xiao-Fan Zhao, Fang Li

**Affiliations:** a College of Chemistry & Chemical Engineering, Shaoxing University, Shaoxing 312000, People’s Republic of China

## Abstract

In the title complex, [CuBr(C_15_H_21_N_2_O)], the Cu^II^ atom is coordinated by one phenolate O, one imine N and one amine N atom of the tridentate Schiff base ligand and by one bromide ion, resulting in a distorted CuBrN_2_O square-planar geometry for the metal ion, with the N atoms in a *cis* conformation.

## Related literature

For a related structure and background references, see the preceding paper: Zhao & Li (2010 ▶).
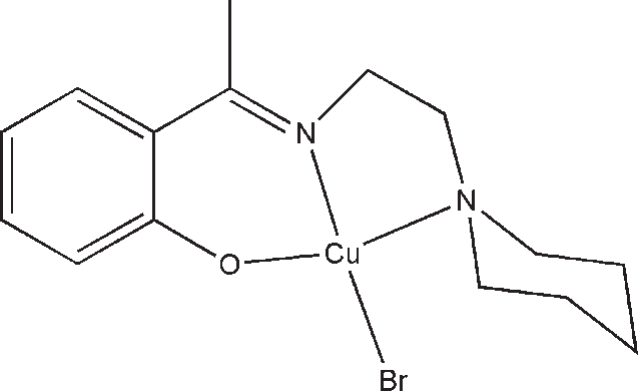


## Experimental

### 

#### Crystal data

[CuBr(C_15_H_21_N_2_O)]
*M* *_r_* = 388.79
Monoclinic, 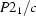

*a* = 10.988 (3) Å
*b* = 17.181 (5) Å
*c* = 8.173 (2) Å
β = 92.366 (3)°
*V* = 1541.6 (7) Å^3^
*Z* = 4
Mo *K*α radiation
μ = 4.01 mm^−1^
*T* = 298 K
0.27 × 0.23 × 0.23 mm

#### Data collection

Bruker SMART CCD diffractometer
Absorption correction: multi-scan (*SADABS*; Sheldrick, 1996 ▶) *T*_min_ = 0.411, *T*_max_ = 0.459
9503 measured reflections
3357 independent reflections
2373 reflections with *I* > 2σ(*I*)
*R*_int_ = 0.044

#### Refinement

*R*[*F* ^2^ > 2σ(*F* ^2^)] = 0.035
*wR*(*F* ^2^) = 0.084
*S* = 1.06
3357 reflections
182 parameters
H-atom parameters constrained
Δρ_max_ = 0.40 e Å^−3^
Δρ_min_ = −0.49 e Å^−3^

### 

Data collection: *SMART* (Bruker, 1998 ▶); cell refinement: *SAINT* (Bruker, 1998 ▶); data reduction: *SAINT*; program(s) used to solve structure: *SHELXS97* (Sheldrick, 2008 ▶); program(s) used to refine structure: *SHELXL97* (Sheldrick, 2008 ▶); molecular graphics: *SHELXTL* (Sheldrick, 2008 ▶); software used to prepare material for publication: *SHELXTL*.

## Supplementary Material

Crystal structure: contains datablocks global, I. DOI: 10.1107/S1600536810026711/hb5544sup1.cif

Structure factors: contains datablocks I. DOI: 10.1107/S1600536810026711/hb5544Isup2.hkl

Additional supplementary materials:  crystallographic information; 3D view; checkCIF report

## Figures and Tables

**Table:** 

Cu1—O1	1.872 (3)
Cu1—N1	1.954 (3)
Cu1—N2	2.034 (3)
Cu1—Br1	2.4030 (7)

**Table:** 

O1—Cu1—N1	91.04 (11)
O1—Cu1—N2	162.99 (11)
N1—Cu1—N2	86.20 (11)
O1—Cu1—Br1	92.26 (8)
N1—Cu1—Br1	159.19 (8)
N2—Cu1—Br1	96.27 (8)

**Fig. 1.**
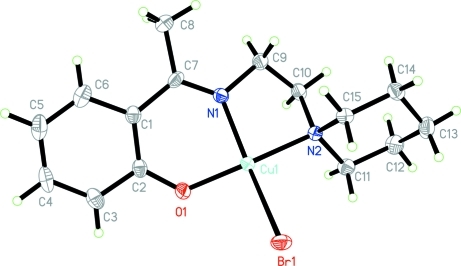
The structure of the title complex, showing 30% displacement ellipsoids (arbitrary spheres for the H atoms).

## supplementary crystallographic information

### Experimental

1-(2-Hydroxyphenyl)ethanone (1 mmol, 136 mg), 2-piperidin-1-ylethylamine (1 mmol, 128 mg), and copper(II) bromide (1 mmol, 223 mg) were dissolved in methanol (80 ml). The mixture was stirred at room temperature for 1 h to give a blue solution. The resulting solution was kept in air for a few days, and blue blocks of (I) were formed.

### Refinement

H atoms were placed in idealized positions and constrained to ride on their parent atoms, with C—H distances in the range 0.93–0.97 Å, and with *U*_iso_(H) = 1.2 or 1.5*U*_eq_(C).

### Crystal data

**Table:**

[CuBr(C_15_H_21_N_2_O)]	*F*(000) = 788
*M**_r_* = 388.79	*D*_x_ = 1.675 Mg m^−^^3^
Monoclinic, *P*2_1_/*c*	Mo *K*α radiation, λ = 0.71073 Å
Hall symbol: -P 2ybc	Cell parameters from 2701 reflections
*a* = 10.988 (3) Å	θ = 2.3–26.3°
*b* = 17.181 (5) Å	µ = 4.01 mm^−^^1^
*c* = 8.173 (2) Å	*T* = 298 K
β = 92.366 (3)°	Block, blue
*V* = 1541.6 (7) Å^3^	0.27 × 0.23 × 0.23 mm
*Z* = 4	

### Data collection

**Table:**

Bruker SMART CCD diffractometer	3357 independent reflections
Radiation source: fine-focus sealed tube	2373 reflections with *I* > 2σ(*I*)
graphite	*R*_int_ = 0.044
ω scans	θ_max_ = 27.0°, θ_min_ = 2.2°
Absorption correction: multi-scan (*SADABS*; Sheldrick, 1996)	*h* = −12→14
*T*_min_ = 0.411, *T*_max_ = 0.459	*k* = −21→21
9503 measured reflections	*l* = −7→10

### Refinement

**Table:**

Refinement on *F*^2^	Primary atom site location: structure-invariant direct methods
Least-squares matrix: full	Secondary atom site location: difference Fourier map
*R*[*F*^2^ > 2σ(*F*^2^)] = 0.035	Hydrogen site location: inferred from neighbouring sites
*wR*(*F*^2^) = 0.084	H-atom parameters constrained
*S* = 1.06	*w* = 1/[σ^2^(*F*_o_^2^) + (0.0318*P*)^2^ + 0.4765*P*] where *P* = (*F*_o_^2^ + 2*F*_c_^2^)/3
3357 reflections	(Δ/σ)_max_ < 0.001
182 parameters	Δρ_max_ = 0.40 e Å^−^^3^
0 restraints	Δρ_min_ = −0.49 e Å^−^^3^

### Special details

**Table:**

Geometry. All e.s.d.'s (except the e.s.d. in the dihedral angle between two l.s. planes) are estimated using the full covariance matrix. The cell e.s.d.'s are taken into account individually in the estimation of e.s.d.'s in distances, angles and torsion angles; correlations between e.s.d.'s in cell parameters are only used when they are defined by crystal symmetry. An approximate (isotropic) treatment of cell e.s.d.'s is used for estimating e.s.d.'s involving l.s. planes.
Refinement. Refinement of *F*^2^ against ALL reflections. The weighted *R*-factor *wR* and goodness of fit *S* are based on *F*^2^, conventional *R*-factors *R* are based on *F*, with *F* set to zero for negative *F*^2^. The threshold expression of *F*^2^ > σ(*F*^2^) is used only for calculating *R*-factors(gt) *etc*. and is not relevant to the choice of reflections for refinement. *R*-factors based on *F*^2^ are statistically about twice as large as those based on *F*, and *R*- factors based on ALL data will be even larger.

### Fractional atomic coordinates and isotropic or equivalent isotropic displacement parameters (Å^2^)

**Table:**

	*x*	*y*	*z*	*U*_iso_*/*U*_eq_	
Cu1	0.50444 (4)	0.13785 (2)	0.50364 (5)	0.02851 (12)	
N1	0.6334 (3)	0.12175 (16)	0.3495 (3)	0.0315 (7)	
N2	0.3861 (2)	0.12461 (14)	0.3077 (3)	0.0257 (6)	
O1	0.6161 (2)	0.17983 (15)	0.6586 (3)	0.0441 (7)	
Br1	0.36084 (4)	0.10855 (2)	0.70885 (4)	0.04276 (13)	
C1	0.8014 (3)	0.13107 (19)	0.5452 (4)	0.0327 (8)	
C2	0.7327 (3)	0.16805 (19)	0.6674 (4)	0.0318 (8)	
C3	0.7973 (4)	0.1946 (2)	0.8102 (5)	0.0428 (9)	
H3	0.7548	0.2208	0.8895	0.051*	
C4	0.9194 (4)	0.1831 (2)	0.8359 (5)	0.0514 (11)	
H4	0.9586	0.2013	0.9314	0.062*	
C5	0.9849 (4)	0.1445 (3)	0.7204 (6)	0.0580 (12)	
H5	1.0677	0.1355	0.7386	0.070*	
C6	0.9266 (3)	0.1197 (2)	0.5789 (5)	0.0498 (11)	
H6	0.9717	0.0942	0.5015	0.060*	
C7	0.7472 (3)	0.1122 (2)	0.3834 (4)	0.0333 (8)	
C8	0.8306 (4)	0.0842 (3)	0.2529 (5)	0.0555 (12)	
H8A	0.7826	0.0659	0.1599	0.083*	
H8B	0.8807	0.0426	0.2959	0.083*	
H8C	0.8815	0.1264	0.2202	0.083*	
C9	0.5824 (3)	0.1139 (2)	0.1796 (4)	0.0401 (9)	
H9A	0.5748	0.0594	0.1503	0.048*	
H9B	0.6357	0.1388	0.1038	0.048*	
C10	0.4607 (3)	0.1519 (2)	0.1714 (4)	0.0354 (9)	
H10A	0.4705	0.2079	0.1781	0.042*	
H10B	0.4192	0.1397	0.0675	0.042*	
C11	0.2726 (3)	0.1720 (2)	0.3175 (4)	0.0365 (9)	
H11A	0.2943	0.2267	0.3228	0.044*	
H11B	0.2327	0.1589	0.4174	0.044*	
C12	0.1837 (4)	0.1588 (2)	0.1714 (5)	0.0478 (10)	
H12A	0.1102	0.1888	0.1863	0.057*	
H12B	0.2202	0.1769	0.0722	0.057*	
C13	0.1513 (4)	0.0739 (3)	0.1531 (5)	0.0514 (11)	
H13A	0.1009	0.0664	0.0542	0.062*	
H13B	0.1050	0.0574	0.2455	0.062*	
C14	0.2659 (3)	0.0248 (2)	0.1448 (4)	0.0368 (9)	
H14A	0.2440	−0.0299	0.1429	0.044*	
H14B	0.3066	0.0365	0.0446	0.044*	
C15	0.3516 (3)	0.04072 (19)	0.2906 (4)	0.0300 (8)	
H15A	0.3132	0.0240	0.3895	0.036*	
H15B	0.4250	0.0100	0.2801	0.036*	

### Atomic displacement parameters (Å^2^)

**Table:**

	*U*^11^	*U*^22^	*U*^33^	*U*^12^	*U*^13^	*U*^23^
Cu1	0.0263 (2)	0.0346 (2)	0.0246 (2)	0.00112 (18)	0.00036 (16)	−0.00404 (17)
N1	0.0309 (18)	0.0366 (17)	0.0272 (16)	−0.0056 (12)	0.0035 (12)	−0.0042 (12)
N2	0.0262 (16)	0.0254 (15)	0.0255 (15)	0.0003 (11)	0.0006 (11)	0.0015 (11)
O1	0.0312 (15)	0.0577 (17)	0.0430 (16)	0.0065 (12)	−0.0044 (11)	−0.0243 (13)
Br1	0.0439 (3)	0.0565 (3)	0.0286 (2)	0.00307 (19)	0.00991 (16)	0.00076 (18)
C1	0.031 (2)	0.0294 (18)	0.037 (2)	−0.0027 (15)	−0.0023 (15)	0.0009 (15)
C2	0.032 (2)	0.0254 (17)	0.038 (2)	−0.0024 (15)	−0.0060 (15)	−0.0011 (16)
C3	0.053 (3)	0.033 (2)	0.041 (2)	−0.0026 (18)	−0.0108 (18)	−0.0011 (17)
C4	0.047 (3)	0.053 (3)	0.052 (3)	−0.011 (2)	−0.025 (2)	0.008 (2)
C5	0.038 (3)	0.072 (3)	0.062 (3)	−0.001 (2)	−0.016 (2)	0.011 (3)
C6	0.032 (2)	0.056 (3)	0.062 (3)	0.0000 (18)	0.001 (2)	0.006 (2)
C7	0.023 (2)	0.0343 (19)	0.044 (2)	−0.0033 (15)	0.0089 (16)	−0.0029 (16)
C8	0.032 (2)	0.083 (3)	0.052 (3)	0.000 (2)	0.0083 (19)	−0.019 (2)
C9	0.033 (2)	0.059 (2)	0.029 (2)	−0.0121 (18)	0.0057 (16)	−0.0005 (18)
C10	0.037 (2)	0.041 (2)	0.0274 (19)	−0.0124 (17)	0.0010 (15)	0.0044 (16)
C11	0.037 (2)	0.034 (2)	0.039 (2)	0.0100 (16)	0.0004 (16)	−0.0004 (17)
C12	0.038 (2)	0.058 (3)	0.047 (2)	0.018 (2)	−0.0096 (18)	0.003 (2)
C13	0.036 (2)	0.074 (3)	0.043 (2)	−0.011 (2)	−0.0067 (18)	0.005 (2)
C14	0.042 (2)	0.036 (2)	0.033 (2)	−0.0079 (17)	0.0008 (16)	−0.0017 (16)
C15	0.034 (2)	0.0250 (18)	0.0307 (19)	0.0006 (15)	0.0042 (15)	0.0024 (15)

### Geometric parameters (Å, °)

**Table:**

Cu1—O1	1.872 (3)	C8—H8A	0.9600
Cu1—N1	1.954 (3)	C8—H8B	0.9600
Cu1—N2	2.034 (3)	C8—H8C	0.9600
Cu1—Br1	2.4030 (7)	C9—C10	1.487 (5)
N1—C7	1.281 (4)	C9—H9A	0.9700
N1—C9	1.482 (5)	C9—H9B	0.9700
N2—C10	1.485 (4)	C10—H10A	0.9700
N2—C11	1.493 (4)	C10—H10B	0.9700
N2—C15	1.495 (4)	C11—C12	1.528 (5)
O1—C2	1.296 (4)	C11—H11A	0.9700
C1—C6	1.405 (5)	C11—H11B	0.9700
C1—C2	1.425 (5)	C12—C13	1.508 (6)
C1—C7	1.464 (5)	C12—H12A	0.9700
C2—C3	1.417 (5)	C12—H12B	0.9700
C3—C4	1.364 (5)	C13—C14	1.520 (5)
C3—H3	0.9300	C13—H13A	0.9700
C4—C5	1.380 (6)	C13—H13B	0.9700
C4—H4	0.9300	C14—C15	1.513 (5)
C5—C6	1.366 (6)	C14—H14A	0.9700
C5—H5	0.9300	C14—H14B	0.9700
C6—H6	0.9300	C15—H15A	0.9700
C7—C8	1.512 (5)	C15—H15B	0.9700
			
O1—Cu1—N1	91.04 (11)	N1—C9—C10	107.9 (3)
O1—Cu1—N2	162.99 (11)	N1—C9—H9A	110.1
N1—Cu1—N2	86.20 (11)	C10—C9—H9A	110.1
O1—Cu1—Br1	92.26 (8)	N1—C9—H9B	110.1
N1—Cu1—Br1	159.19 (8)	C10—C9—H9B	110.1
N2—Cu1—Br1	96.27 (8)	H9A—C9—H9B	108.4
C7—N1—C9	121.3 (3)	N2—C10—C9	110.6 (3)
C7—N1—Cu1	127.4 (2)	N2—C10—H10A	109.5
C9—N1—Cu1	111.1 (2)	C9—C10—H10A	109.5
C10—N2—C11	110.8 (3)	N2—C10—H10B	109.5
C10—N2—C15	112.3 (2)	C9—C10—H10B	109.5
C11—N2—C15	108.8 (3)	H10A—C10—H10B	108.1
C10—N2—Cu1	101.6 (2)	N2—C11—C12	112.7 (3)
C11—N2—Cu1	113.9 (2)	N2—C11—H11A	109.1
C15—N2—Cu1	109.4 (2)	C12—C11—H11A	109.1
C2—O1—Cu1	126.6 (2)	N2—C11—H11B	109.1
C6—C1—C2	117.8 (3)	C12—C11—H11B	109.1
C6—C1—C7	120.3 (3)	H11A—C11—H11B	107.8
C2—C1—C7	121.6 (3)	C13—C12—C11	111.2 (3)
O1—C2—C3	117.1 (3)	C13—C12—H12A	109.4
O1—C2—C1	125.6 (3)	C11—C12—H12A	109.4
C3—C2—C1	117.4 (3)	C13—C12—H12B	109.4
C4—C3—C2	122.4 (4)	C11—C12—H12B	109.4
C4—C3—H3	118.8	H12A—C12—H12B	108.0
C2—C3—H3	118.8	C12—C13—C14	110.4 (3)
C3—C4—C5	120.1 (4)	C12—C13—H13A	109.6
C3—C4—H4	119.9	C14—C13—H13A	109.6
C5—C4—H4	119.9	C12—C13—H13B	109.6
C6—C5—C4	119.3 (4)	C14—C13—H13B	109.6
C6—C5—H5	120.4	H13A—C13—H13B	108.1
C4—C5—H5	120.4	C15—C14—C13	110.7 (3)
C5—C6—C1	122.9 (4)	C15—C14—H14A	109.5
C5—C6—H6	118.5	C13—C14—H14A	109.5
C1—C6—H6	118.5	C15—C14—H14B	109.5
N1—C7—C1	121.6 (3)	C13—C14—H14B	109.5
N1—C7—C8	120.2 (3)	H14A—C14—H14B	108.1
C1—C7—C8	118.2 (3)	N2—C15—C14	113.3 (3)
C7—C8—H8A	109.5	N2—C15—H15A	108.9
C7—C8—H8B	109.5	C14—C15—H15A	108.9
H8A—C8—H8B	109.5	N2—C15—H15B	108.9
C7—C8—H8C	109.5	C14—C15—H15B	108.9
H8A—C8—H8C	109.5	H15A—C15—H15B	107.7
H8B—C8—H8C	109.5		
